# Near-Infrared Spectroscopy Detection of Off-Flavor Compounds in Tench (*Tinca tinca*) After Depuration in Clean Water

**DOI:** 10.3390/foods14050739

**Published:** 2025-02-21

**Authors:** Daniel Martín-Vertedor, Juan Carlos Ramírez-López, Ricardo S. Aleman, Elisabet Martín-Tornero, Ismael Montero-Fernández

**Affiliations:** 1 Aquaculture Center ‘Las Vegas del Guadiana’, Regional Government of Extremadura, N-5, km 391.7, Villafranco del Guadiana, 06195 Badajoz, Spain; daniel.martin@juntaex.es (D.M.-V.); juancarlos.ramirez@gpex.es (J.C.R.-L.); 2 Research Institute of Agricultural Resources (INURA), University of Extremadura, Avda de la Investigación, s/n, 06006 Badajoz, Spain; ismonterof@unex.es; 3 School of Nutrition and Food Sciences, Louisiana State University Agricultural Center, Baton Rouge, LA 70802, USA; rsantosaleman@lsu.edu; 4 Department of Analytical Chemistry, University of Extremadura, 06006 Badajoz, Spain; 5 Department of Plant Biology, Ecology and Earth Sciences, Faculty of Science, University of Extremadura, Avda. de Elvas, s/n, 06071 Badajoz, Spain

**Keywords:** tench, quality, volatile organic compounds, aromas, NIRS

## Abstract

Tench (*Tinca tinca*) is a warm-temperate, freshwater benthic fish with often unpleasant odors and flavors which result from its natural habitat. These characteristics may deter consumers; therefore, their removal would enhance the fish’s palatability and market appeal. Thus, tench were grown in an aquaculture center and subjected to a clean water depuration system in which six sampling points were carried out at 0 h, 12 h, 24 h, 48 h, 72 h, and 96 h. An analysis was conducted using gas chromatography–mass spectrometry and near-infrared spectroscopy (NIRS), revealing acid derivatives as the predominant families of volatile organic compounds (VOCs). The main off-flavor VOCs were 3,5,5-trimethyl-1-hexene, dimethyl-8-hydronaphtalen, 1-octen-3-ol, diethyl phthalate, 2-methylisoborneol, and a-isomethylionone. Maximum concentrations were observed at 0 h, exceeding 300 μg/g for diethyl phthalate and being less than 55 μg/g for the remaining VOCs. The content progressively decreased from that point on. The spectra obtained by NIRS highlighted differences between the cleaning depuration treatments, exhibiting discrimination among the samples studied (PC1 = 77.8%; PC2 = 11.3%). Finally, dimethyl-8-hydronaphtalen and 2-methylisoborneol were linearly correlated with NIRS data, with $R_{CV}^{2}\;$ values of 0.94 and 0.96, respectively, and RMSECV values of 1.00 and 3.62 μg/g, respectively. Therefore, a clean water depuration system is appropriate to obtain fish with fewer off-flavor characteristics. Moreover, NIRS represents an accurate, inexpensive, and non-destructive technique to determine the optimal time for the water depuration of fish.

## 1. Introduction

Aquaculture is a sector of food production that has experienced the greatest growth worldwide thanks to scientific progress and technological innovation, providing high-quality proteins and fats, and helping achieve sustainable development goals [1]. These foods are needed to provide a healthy and nutritious diet for an ever-growing population. However, some production techniques can lead to conditions that cause fish to acquire undesirable flavors and odors, commonly referred to as ‘off-flavors’ [2]. This problem affects freshwater species in particular, such as rainbow trout (*Oncorhynchus mykiss*), tilapia (*Oreochromis niloticus* and *O. aureus*), largemouth bass (*Micropterus salmoides*), Atlantic salmon (*Salmo salar*), tench (*Tinca tinca*), and others [3] grown in recirculation aquaculture systems (RAS), ponds, cages, and other culture systems.

Tench (*Tinca tinca* L., 1758) is a freshwater cyprinid fish species of Eurasian origin [4]. Its distribution is worldwide, and due to human-mediated translocations [5], it is currently established on every continent except Antarctica [6]. It is an important gastronomic and angling fish, highly valued in local regions of Spain and Italy as well as in European countries such as Poland, Germany, and the Czech Republic [7], although production in 2022 did not reach 700 tons in Europe and barely exceeded 14 tons in Spain [8]. This species is considered one of the best candidates for the diversification of freshwater aquaculture in Europe because it is a traditional crop with high consumer demand and a high market price (between EUR 16 and 18 per kg^−1^) [8]. In addition, it has important natural and cultural value since it has been traditionally reared in extensive monoculture natural ponds frequently associated with other uses of water related to agricultural or livestock purposes [9].

Freshwater species, such as tench or even rainbow trout, live in shallow areas of lakes and ponds, preferring muddy or sandy substrates with submerged vegetation that favors their reproduction [10]. These species are consequently marketed with unpleasant odors and flavors brought about by certain VOCs caused by their environment [3]. Although water recirculation systems are established in aquaculture, they are affected by the presence of phosphorus, micronutrients, and organic matter, which can affect the water quality. These compounds are linked to the growth in phytoplankton that can produce secondary metabolites responsible for off-flavor characteristics in fish but have no negative impacts on human health [11].

Researchers have shown derivatives of alga, fungi, cyanobacteria, and microorganisms such as *Actinomycetales* and *Myxococcales* to play a role in the synthesis of secondary metabolites that are responsible for the unpleasant odor, taste, and color of the final product. The main sensory attributes in aquaculture fish are described as earthy, muddy, or moldy [11], and the primary contributors to these abnormal aromas are tepenoids, carotenoid derivatives, fatty acids, and sulfur compounds [12,13]. Off-flavor aromas resulting from VOCs include 2-methylisoborneol, geosmin, 2-isopropyl-3methoxypurazine, and 2-isobutyl-3-methoxypyrazine [14]. Geosmin and 2-methylisoborneol, in particular, are responsible for unpleasant odors in rainbow trout, tilapia, Arctic charr, largemouth bass, and Atlantic salmon [15]. Therefore, marketing fish with unpleasant flavors and odors has significant commercial implications.

Lipophilic compounds are removed via passive diffusion through the gills or skin, or through the metabolism of the fish, before they are excreted; however, the relative importance of these processes is unknown [2]. There are various techniques for removing the concentration of off-flavor compounds. Some focus on preventing their occurrence by controlling aquatic microorganisms in the culture water by applying ozone or algaecides [2]. However, the simplest method that has been used for centuries is the depuration of fish in clean water for several days or weeks [16]. The duration of this depuration process will depend on the initial concentration of off-flavor compounds in the fish, the species and size of the fish, the volume of water used, and the water temperature [3].

Different methodologies exist for the identification of organic compounds present in fish, but the most widespread is gas chromatography coupled with mass spectrometry with different strategies of extraction techniques [17]. This equipment is widely used to quantify the presence of VOCs, using calibration curves of the corresponding standards. However, few published studies exist on the use of near-infrared spectroscopy (NIRS) as a method to detect abnormal aromatic compounds. Zhou et al. [18] evaluated the freshness of bighead carp (*Aristichthys nobilis*) using this technology along with the multivariate regression method, thereby demonstrating its feasibility for assessing fish freshness. The sensory quality of rainbow trout and other fish species was monitored using NIR spectroscopy in raw fish as a rapid tool to control food quality [19]. Ritthiruangdej and Suwonsichon [20] assessed commercial Thai fish sauces based on NIRS data and chemometric tools. Thus, NIRS could be used as a rapid method to ascertain the amount of unpleasant fish aroma before they are marketed. Therefore, the objective of this study was to apply a washing technique to tench to remove off-flavor compounds, and the results were determined using instrumental and NIRS methods.

## 2. Materials and Methods

### 2.1. Samples

**Figure 1:**
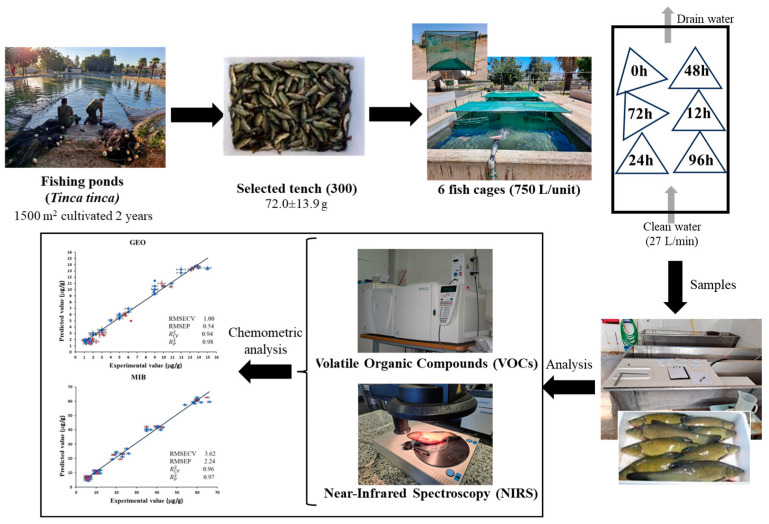
Diagram of experimental design.

This study was carried out at the facilities of the ‘Las Vegas del Guadiana’ Aquaculture Center of the Regional Government of Extremadura, located in Villafranco del Guadiana (Badajoz, Spain) in July 2024. Tench had been reared for two years in 1500 m^2^ earthen bottom ponds at the aquaculture center. At the end of this period, a total of 300 tench were selected at random from the breeding pond. These fish had a mean weight of 72.0 ± 13.9 g. They were divided into six fish cages with a 725 L capacity, with 50 tench in each cage. All cages were placed in a single concrete depuration pond of 35 m^3^ with a renewal rate of 27 L/min of clean water. The water temperature of the rearing pond was 26.1 ± 1.0 °C, while that of the depuration pond was 24.2 ± 1.0 °C. The quality of the water was measured, ensuring it maintained a constant level of dissolved oxygen of 99.05%, a salinity content of 583.8 mg/L, a pH of 7.6, and conductivity of 1164.8 µS/cm. Fish were selected randomly in batches of 10 for the different experimental samples at 0 h, 12 h, 24 h, 48 h, 72 h, and 96 h in triplicate. They were gathered in a bucket with 80 L of water taken from the depuration pond. Next, the fish were killed by a blunt blow to the head in compliance with the animal euthanasia method defined in Annex III, Section 1.b of the Spanish Royal Decree 53/2013. Furthermore, this study was carried out under the 148b/2020 authorization of the project titled “Comparative study of the growth and other biometric aspects and susceptibility to parasites in mixed and monosex culture of tench”. Finally, the fish were weighed and frozen at −20 °C until the analysis was conducted. The cage and unused fish were returned to the purification pond until the end of the experiment. No supplemental feeding was given during this period. The diagram of the experimental design is shown in Figure 1.

### 2.2. Analysis of VOCs in Tench

The VOC analysis was carried out using the headspace method described by Sánchez et al. [21]. Two grams of tench tissue (*longissimus dorsi*) were introduced into a vial containing 7 mL of NaCl solution (30% *w*/*v,* Merck, KGaA, Darmstadt, Germany). A polydimethylsiloxane/divinylbenzene (PDMS/DVB) StableFlex fiber (65 μm, Supelco^TM^ Analytical, Bellefonte, PA, USA) was used to absorb VOCs from the samples. The prepared samples were incubated at 40 °C for 30 min. After that, the fiber was inserted for desorption at 250 °C for 15 min into the injection port of the gas chromatograph with a triple quadrupole mass spectrometry detector (model 456-GC) and with an Agilent DB WAXetr capillary column (60 m × 0.25 mm; DI: 0.25 mm). The detected peaks were identified using the NIST 2.0 MS reference spectral library. Compound identification was carried out by comparing their mass spectra and linear retention indexes (LRIs) with those of the injected standards (Sigma-Aldrich, St. Louis, MO, USA) or with the mass spectra contained in the NIST standard reference database. The quantitative analysis was performed by area comparison using an internal standard (2-octanol) of known concentration.

### 2.3. Near-Infrared Spectroscopy Analysis

Infrared spectra were registered with a near-infrared spectrometer (Agilent Cary Series UV-Vis-NIRS Spectrophotometer, Santa Clara, CA, USA) in absorbance mode. The spectrometer is equipped with a Xenon flash lamp (250 Hz) with a photometric system with double beam power. The spectrum of each sample was obtained within a wavelength of 400–2400 nm. The spectral resolution was 0.2 nm, and the number of spectra at each sampling point was 80. A Teflon sample was used as the reference blank. The fish was placed on a rotating Petri dish and measured at different points for each fish. The readings were conducted in triplicate, and the average of these spectra was used for subsequent analyses.

### 2.4. Multivariate Data Analysis

Principal component analysis (PCA) was applied to observe the discrimination obtained by NIRS. Partial Least Squares (PLS) methods were performed to obtain calibration models to quantify the VOCs. To build the quantification models, the sample set was first randomly divided into two groups. The calibration set, which accounted for 70% of the samples, was used for calibration and cross-validation. Meanwhile, the remaining 30% (test set) was used to evaluate the accuracy and robustness of the models.

To evaluate the models’ performance, several statistical indicators were employed, including the determination coefficient (R^2^), root mean square error of cross-validation (RMSECV), and relative error of prediction (REP).

The program used for the multivariate analysis was Matlab R2016b, version 9.1 (Mathworks Inc., Natick, MA, USA) with PLS_Toolbox 8.2.1 (Eigenvector Research Inc., Wenatchee, WA, USA).

## 3. Results and Discussion

### 3.1. Effect of Water Depuration on Volatile Organic Compounds

Table 1 presents the main families of VOCs in fish samples subjected to different water depuration times. Acid derivatives were the most abundant, while phenols, ketones, alcohols, hydrocarbons, and terpenes were present in smaller proportions.

Some VOCs increase with longer fish washing times, as removing off-flavor aromatic compounds during washing may enhance the detection of certain compounds with pleasant aromas that intensify with increased washing.

This applies to phenol, alcohol, and hydrocarbon families, which are compounds responsible for positive aromas. The phenol group doubled after 48 h of sample washing. Additionally, after 24 h of water depuration, alcohol groups increased significantly. These aromas are related to a fish-like, green, citrus, or fruity odor [22]. Hydrocarbons were the second most important VOCs, showing an increase of 20% in fish subjected to longer washing times compared with fish submitted to no washing treatment (0 h). As per Maggi et al. [23], hydrocarbons may result from the degradation of fatty acids, contributing to a sweet flavor.

By contrast, families of VOCs such as ketones, terpenes, and acid derivatives were found in greater proportions in tench that spent less time in washing ponds (0 h, 12 h, and 24 h), with levels being 10–20% higher the in initial treatments. Acid derivatives were the most represented VOCs throughout the treatments. However, a significant aroma reduction occurred after 24 h, and content levels equalized from 48 h onward.

**Figure 2:**
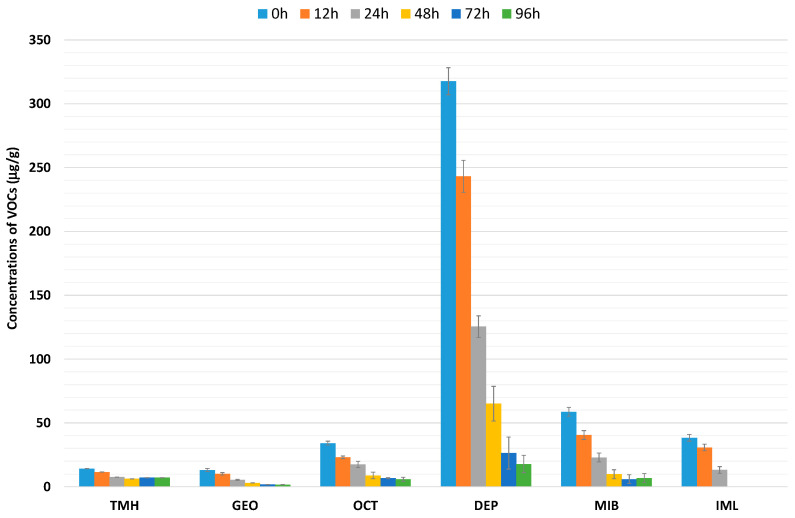
Concentrations of VOCs (μg/g) obtained in tench during washing treatments. TMH: 3,5,5-trimethyl-1-hexene; GEO: dimethyl-8-hydronaphtalen; OCT: 1-octen-3-ol; DEP: diethyl phthalate; MIB: 2-methylisoborneol; IML: α-isomethylionone.

Figure 2 shows the main VOCs associated with off-flavor aromas, namely 3,5,5-trimethyl-1-hexene (TMH), dimethyl-8-hydronaphtalen (GEO), 1-octen-3-ol (OCT), diethyl phthalate (DEP), 2-methylisoborneol (MIB), and α-isomethylionone (IML).

The highest VOC concentrations (Figure 2) were observed in the treatment at 0 h, and it gradually decreased in all fish subjected to the higher-intensity depuration process with clean water. In fact, after 24 h, these VOCs decreased by 50%, continuing to drop after 48 h, with some compounds such as α-isomethylionone showing non-detectable levels. The key VOCs responsible for off-flavor aromas, as highlighted by the existing literature, are dimethyl-8-hydronaphtalen, also known as geosmin, and 2-methylisoborneol [3]. Although not present in high concentrations, these compounds play a crucial role in the unpleasant aroma associated with freshwater fish, particularly benthic species like tench. Notably, diethyl phthalate had the highest concentration in this study, peaking in the washing treatments at 0 h. Diethyl phthalate is a phthalic acid ester commonly used in plastics or cosmetics, has a characteristic unpleasant odor, and is recognized as a pollutant [24].

In the samples, 1-octen-3-ol, an unsaturated alcohol characterized by its contribution of a ‘mushroom-like’ and ‘raw mushroom’ flavor [25] and that is synthesized by different species of microorganisms [22], was identified. Iglesias et al. [26] demonstrated that 1-octen-3-ol is associated with the fat oxidation index of fish, which can contribute to the negative odor characteristic of this substance. Furthermore, low concentrations were recorded of 3,5,5-trimethyl-1-hexene, a compound associated with an earthy smell [27]. These results indicate that fish subjected to depuration with clean water for more than 24 h before slaughter show significantly reduced levels of certain VOCs that contribute to unpleasant odors.

Therefore, this study demonstrates that a clean water purification system can dilute the compounds responsible for off-flavors, resulting in tench with a lower negative aromatic profile. Thus, these findings support the potential to offer tench with better sensory characteristics, potentially increasing the consumption of this product.

### 3.2. Evaluation of NIRS Spectra Data

**Figure 3:**
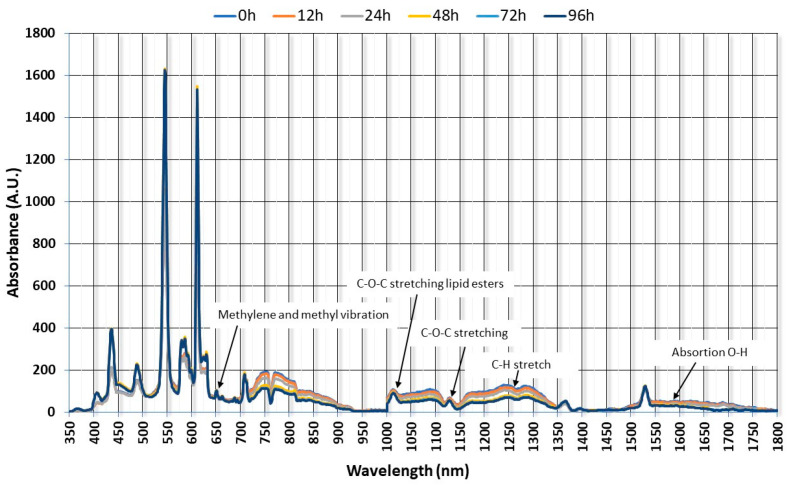
NIR spectra obtained from fish samples after application of different clean water depuration processes.

Figure 3 shows the average spectrum of each batch of 10 fish after being subjected to depuration with clean water at 0 h, 12 h, 24 h, 48 h, 72 h, and 96 h. The initial and final bands of the infrared spectrum have not been taken into account since absorptions of the functional groups were not observed in those characteristic regions. The main peaks originate from the stretching vibrations of -CH, -OH, and -NH groups. Thus, the characteristic bands of the asymmetric stretching of the methyl groups (-CH_3_) are observed at 400 nm, while the asymmetric and symmetric stretching of the methylene groups appear at 362 nm and 374 nm, respectively. The C-H absorption bands of the aliphatic chains of fatty acids are also observed at 440 nm.

Another characteristic band, corresponding to the carbonyl group found in aldehydes, ketones, and esters, appears at 578 nm (Figure 3). Methylene and methyl scissor vibrations are observed at 685.1 nm, and the asymmetric C-O-C stretching of lipid esters is observed at 1020 nm. Other lipid-related vibrations present in the fish were methylene bending and C-O stretching and are observed at 1125 nm, while symmetric PO_4_^2−^ stretching along with lipid (C-C) and C-O vibrations appear at 900 nm and 880 nm [21,22]. Other key lipid absorbance peaks are C-H and CH_2_ vibrations using FT-NIR spectroscopy. These signals, related to the fatty acid content, appear around 1680–1710 nm [28,29,30]. The first overtone of the C-H stretching is observed in the range of 770–998 nm, and the second overtone between 719 and 900 nm. At around 1150–1200 nm, a band can be seen that is the harmonic of the C-H groups of lipid methylenes. This is associated with the second overtone of the C-H bonds that include the CH, CH_2_, and CH_3_ groups. Finally, the bands at 890 and 940 nm correspond to the third harmonic of the C-H stretching of saturated and unsaturated fats [31]. At around 720 nm, the first harmonic of the O-H group’s stretching is observed. This is attributed to water absorption bands, as the samples analyzed were fresh fish. These bands also overlap with those of the C-H group’s stretching and C-H deformations that correspond to -CH_2_ and -CH_3_ bonds [32]. The stretching of N-H bonds due to proteins is also observed at 760 nm. At around 1528 nm, we can see thin, sharp bands that are responsible for the N-H bonds of protein molecules [33,34].

Figure 3 shows three key regions where differences between spectra occurred with the washing time, classified as 400–650 nm, 700–900 nm, and 1000–1350 nm, respectively. Absorbance was lowered as the washing time increased. It is in these regions that the absorption bands of the characteristic families of VOCs included in Figure 2 produced significant differences in the absorption intensity.

### 3.3. NIRS Discrimination Samples After the Application of Different Clean Water Depuration Processes

**Figure 4:**
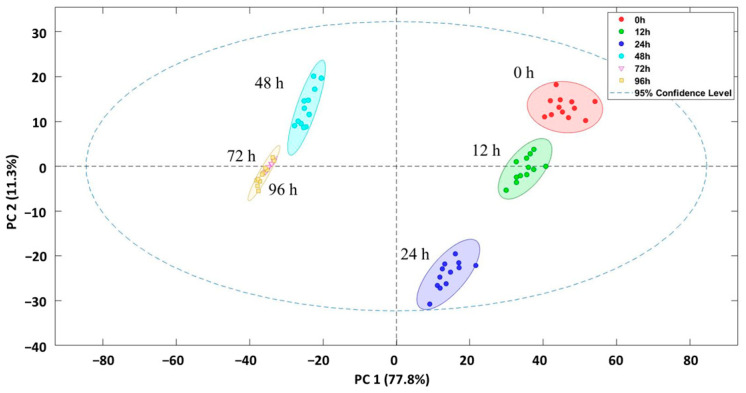
Principal component analysis of fish samples after application of different clean water depuration processes at 0 h, 12 h, 24 h, 48 h, 72 h, and 96 h.

Given the differences observed in the NIRS spectra of the samples subjected to different washing times, an exploratory analysis using PCA was performed to determine if the samples could be differentiated. The results of the absorption signals obtained by NIRS are displayed in Figure 4. The score plot shows that principal component 1 (PC1) explained 77.8% of the cases and principal component 2 (PC2) explained 11.3%, with a total of 89.1% of the cases being explained at a 95.0% confidence level. There are five clusters, with all sample groups separated except for those washed for 72 and 96 h, which are indistinguishable in the PCA and grouped in the same cluster.

Control samples (0 h) and 12 and 24 h samples are on the positive side of PC1, while those at 48 h, 72 h, and 96 h appear on the negative side. The treatment at 48 h already exhibits notable differences in VOCs in relation to the other treatments, indicating that the profile of the samples recorded by NIRS differed with an increased washing time.

Therefore, it can be concluded that NIRS is capable of discriminating fish samples washed extensively from those washed for a shorter period or not at all.

Agyekum et al. [35] previously applied the NIRS technique coupled with chemometric algorithms to predict fish freshness. Correlations greater than 95% were obtained, highlighting this technique’s potential as a quality indicator in the food industry. Furthermore, chemometric models, like those developed by Grassi et al. [36], have shown prediction accuracies of up to 100% in some cases, demonstrating the utility of this method in combating fraud in the fish industry. Recent studies have also confirmed that this technique provides good prediction models for the control of toxic substances such as histamine in tuna fish [37].

### 3.4. Prediction of Dimethyl-8-Hydronaphtalen and 2-Methylisoborneol Using NIRS Technology

**Figure 5:**
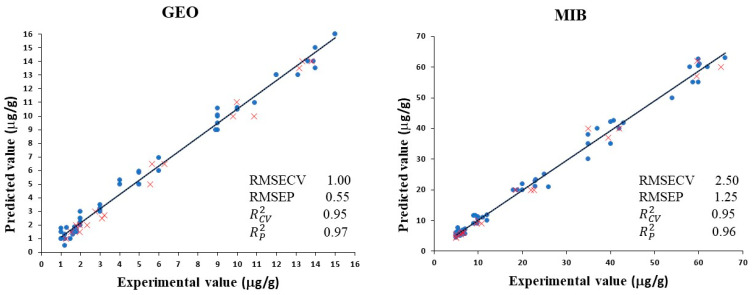
PLS cross-validation predictions (●) and validation set predictions (x) for GEO: dimethyl-8-hydronaphtalen and MIB: 2-methylisoborneol quantified (μg/g) and their correlations with NIRS data.

To harness the potential of the NIRS technique in analyzing compounds after clean water purification, a Partial Least Squares (PLS) algorithm was employed to develop quantification models to predict the content of the main off-flavor compounds. PLS models were built between the concentration of dimethyl-8-hydronaphtalen and 2-methylisoborneol and analyzed using the reference method and the data obtained using NIRS technology. The outcomes are shown in Figure 5.

A calibration set was used to select the optimum number of components and build the PLS model. Two different PLS models were built, one for each VOC. Eight components were required to obtain the model for GEO and MIB, explaining 94% and 96% of the total variance, respectively.

A strong linear correlation can be seen between the concentration of VOCs obtained by the reference method and the data obtained with the near-infrared equipment. The $R_{CV}^{2}\;$ values for the two models were 0.94 and 0.96, respectively, with RMSECV values of 1.00 and 3.62 μg/g. Furthermore, the model was validated with tench samples subjected to different water purification methods. The validation results show strong model performance, with an $R_{P}^{2}\;$ value of 0.98 for dimethyl-8-hydronaphtalen and 0.97 for 2-methylisoborneol. The RMSEP values were 0.54 and 2.24 μg/g, respectively. These values confirm that NIR spectroscopy combined with PLS can accurately predict GEO and MIB in this species of fish.

It can determine the optimal point at which tench present the minimum levels of off-flavor aroma so that they may be marketed with the highest quality. To the best of our knowledge, no such specific studies have verified the off-flavor compounds of fish with NIRS equipment. However, our study aligns with previous studies in which a Partial Least Squares regression with NIRS data and other chemical parameters were used to evaluate food quality and safety. Cheng et al. [38], for instance, demonstrated NIRS to be a rapid method to predict the chemical properties of fish muscle. NIRS also successfully predicted the freshness of fish at the initial stage of decomposition [35]. NIRS combined with a PLS algorithm were used to assess fish spoilage during a period of 12 days in low-temperature storage [39]. Sanhueza et al. [40] proposed a multivariate analysis using NIRS for the characterization of sardine, silverside, and anchovy to quantify freshness.

Given our findings and the results obtained by other researchers, it would be beneficial to incorporate a processing line with NIRS equipment that allows aquaculture industry professionals to predict the content of off-flavor aromatic compounds in fish. This would enable them to assess the need for a purification system prior to slaughter.

## 4. Conclusions

The use of a clean water purification system before marketing fish significantly reduces the content of off-flavor compounds. Further reductions are observed as the washing time increases. NIR spectroscopy is capable of discriminating fish samples subjected to different purification times in clean water. Coupled with a chemometric method, this technology shows great potential as a reliable and efficient tool to detect off-flavor compounds in tench. Aquaculture companies should make use of such equipment on site given that it is rapid, non-invasive, and environmentally friendly. Operating this equipment is easy and simply requires a technician to interpret the data and make informed decisions based upon them. Implementing this technology could facilitate the detection of these abnormal substances, ensuring that tench with superior sensory qualities reach the market. An improvement in product quality is likely to increase consumer demand and boost the appeal of tench as a valuable aquaculture product. For future research, we will evaluate the possibility of repeating this study with other commercially relevant native species from the main basins of the Iberian Peninsula.

## Figures and Tables

**Table 1:** Distribution of VOC families in tench samples (%).

	0 h	12 h	24 h	48 h	72 h	96 h
Hydrocarbons	10.7 ± 1.3	12.5 ± 1.1	12.1 ± 1.0	16.7 ± 1.2	15.4 ± 1.5	15.8 ± 1.6
Alcohols	8.5 ± 1.4	7.8 ± 1.1	12.7 ± 0.3	17.9 ± 1.3	18.8 ± 1.6	21.4 ± 2.2
Acid derivates	59.1 ± 2.1	58.7 ± 1.1	54.6 ± 3.2	48.5 ± 2.6	47.7 ± 3.8	45.1 ± 5.4
Terpenes	12.5 ± 1.0	11.1 ± 1.3	11.5 ± 1.3	9.9 ± 1.1	9.0 ± 1.2	9.5 ± 2.1
Ketones	6.9 ± 2.1	7.7 ± 1.0	6.8 ± 1.5	2.1 ± 0.3	4.8 ± 0.6	4.1 ± 0.6
Phenols	2.3 ± 0.6	2.2 ± 0.5	2.1 ± 0.5	4.7 ± 1.2	4.1 ± 1.1	3.8 ± 0.6

## Data Availability

The original contributions presented in the study are included in the article, further inquiries can be directed to the corresponding author.

## References

[1] FAO (2022). El Estado Mundial de la Pesca y la Acuicultura 2022. Hacia la Transformación Azul.

[2] Tucker C.S. (2000). Off-flavor problems in aquaculture. Rev. Fish. Sci..

[3] Moretto J.A., Freitas P.N., Souza J.P., Oliveira T.M., Brites I., Pinto E. (2022). Off-flavors in aquacultured fish: Origins and implications for consumers. Fishes.

[4] Kottelat M., Freyhof J. (2007). Handbook of European Freshwater Fishes.

[5] Lajbner Z., Linhart O., Kotlík P. (2011). Human-aided dispersal has altered but not erased the phylogeography of the tench: Human-aided dispersal in fish phylogeography. Evol. Appl..

[6] Avlijaš S., Ricciardi A., Mandrak N.E. (2018). Eurasian tench (*Tinca tinca*): The next Great Lakes invader. Can. J. Fish. Aquat. Sci..

[7] Fernández I., Larrán A.M., de Paz P., Riesco M.F. (2024). The direct effects of climate change on tench (*Tinca tinca*) sperm quality under a real heatwave event scenario. Animals.

[8] Asociación Empresarial de Acuicultura de España (APROMAR) (2024). Report on Aquaculture in Spain 2024.

[9] Pula H.J., Trenzado C.E., García-Mesa S., Fallola C., Sanz A. (2018). Effects of different culture systems on growth, immune status, and other physiological parameters of tench (*Tinca tinca*). Aquaculture.

[10] Rendón P.M., Gallardo J.M., Ceballos E.G., Regadera J.J.P., García J.C.E. (2003). Determination of substrate preferences of Tench, *Tinca tinca* (L.), under controlled experimental conditions. J. Appl. Ichthyol..

[11] Freeman K.S. (2010). Harmful algal blooms: Musty warnings of toxicity. Environ. Health Perspect..

[12] Lukassen M.B., de Jonge N., Bjerregaard S.M., Podduturi R., Jørgensen N.O., Petersen M.A., Gianmarco S.D., da Silva R.J., Nielsen J.L. (2019). Microbial production of the off-flavor geosmin in tilapia production in Brazilian water reservoirs: Importance of bacteria in the intestine and other fish-associated environments. Front. Microbiol..

[13] Li Z., Hobson P., An W., Burch M.D., House J., Yang M. (2012). Earthy odor compounds production and loss in three cyanobacterial cultures. Water Res..

[14] Olsen B.K., Chislock M.F., Wilson A.E. (2016). Eutrophication mediates a common off-flavor compound, 2-methylisoborneol, in a drinking water reservoir. Water Res..

[15] Podduturi R., Petersen M.A., Vestergaard M., Jørgensen N.O. (2020). Geosmin fluctuations and potential hotspots for elevated levels in recirculated aquaculture system (RAS): A case study from pikeperch (*Stizostedion lucioperca*) production in Denmark. Aquaculture.

[16] Bonpunt E. (2018). The off-flavors management in the production of farmed sturgeon. The Siberian Sturgeon (Acipenser baerii, Brandt, 1869) Volume 2—Farming.

[17] Adebo O.A., Oyeyinka S.A., Adebiyi J.A., Feng X., Wilkin J.D., Kewuyemi Y.O., Abrahams A.M., Tugizimana F. (2021). Application of gas chromatography–mass spectrometry (GC-MS)-based metabolomics for the study of fermented cereal and legume foods: A review. Int. J. Food Sci. Technol..

[18] Zhou J., Wu X., Chen Z., You J., Xiong S. (2019). Evaluation of freshness in freshwater fish based on near infrared reflectance spectroscopy and chemometrics. LWT.

[19] Warm K., Martens H., Nielsen J., Martens M. (2001). Sensory quality criteria for five fish species predicted from near-infrared (NIR) reflectance measurement. J. Food Qual..

[20] Ritthiruangdej P., Suwonsichon T. (2007). Relationships between NIR spectra and sensory attributes of Thai commercial fish sauces. Anal. Sci..

[21] Sánchez R., Martín-Tornero E., Lozano J., Fernández A., Arroyo P., Meléndez F., Martín-Vertedor D. (2022). Electronic nose application for the discrimination of sterilization treatments applied to Californian-style black olive varieties. J. Sci. Food Agric..

[22] de Carvalho D.S., Dionísio A.P., dos Santos R., Boguzs S., Godoy H.T., Pastore G.M. (2011). Production of 1-octen-3-ol by Neurospora species isolated from beiju in different culture medium. Procedia Food Sci..

[23] Maggi F., Papa F., Cristalli G., Sagratini G., Vittori S. (2010). Characterisation of the mushroom-like flavour of *Melittis melissophyllum* L. subsp. *melissophyllum* by headspace solid-phase microextraction (HS-SPME) coupled with gas chromatography (GC–FID) and gas chromatography–mass spectrometry (GC–MS). Food Chem..

[24] Lu Y., Tang F., Wang Y., Zhao J., Zeng X., Luo Q., Wang L. (2009). Biodegradation of dimethyl phthalate, diethyl phthalate and di-n-butyl phthalate by *Rhodococcus* sp. L4 isolated from activated sludge. J. Hazard. Mater..

[25] Xiao Z., Liu L., Niu Y., Zhang J., Wang D., Zhou C. (2024). Mushroom alcohol (1-octen-3-ol) and other 7 aroma compounds selected from Chinese dry-cured hams can enhance saltiness perception. Meat Sci..

[26] Iglesias J., Medina I. (2008). Solid-phase microextraction method for the determination of volatile compounds associated to oxidation of fish muscle. J. Chromatogr. A.

[27] Li Y., Yuan L., Liu H., Liu H., Zhou Y., Li M., Gao R. (2023). Analysis of the changes of volatile flavor compounds in a traditional Chinese shrimp paste during fermentation based on electronic nose, SPME-GC-MS and HS-GC-IMS. Food Sci. Hum. Wellness.

[28] Zhang Q., Liu C., Sun Z., Hu X., Shen Q., Wu J. (2012). Authentication of edible vegetable oils adulterated with used frying oil by Fourier Transform Infrared Spectroscopy. Food Chem..

[29] Socrates G. (2004). Infrared and Raman Characteristic Group Frequencies: Tables and Charts.

[30] Aenugu H.P.R., Kumar D.S., Srisudharson N.P., Ghosh S., Banji D. (2011). Near infrared spectroscopy—An overview. Int. J. ChemTech Res..

[31] Panagou E.Z., Papadopoulou O., Carstensen J.M., Nychas G.-J.E. (2014). Potential of multispectral imaging technology for rapid and non-destructive determination of the microbiological quality of beef filets during aerobic storage. Int. J. Food Microbiol..

[32] Workman J., Weyer L. (2007). Practical Guide to Interpretive Near-Infrared Spectroscopy.

[33] Khodabux K., L’Omelette M.S.S., Jhaumeer-Laulloo S., Ramasami P., Rondeau P. (2007). Chemical and near-infrared determination of moisture, fat and protein in tuna fishes. Food Chem..

[34] Osborne B.G. (2006). Near infrared spectroscopy in food analysis. Encyclopedia of Analytical Chemistry: Applications, Theory and Instrumentation.

[35] Agyekum A.A., Hutsanedzie F.Y.H., Annavaram V., Mintah B.K., Asare E.K., Wang B. (2020). FT-NIR coupled chemometric methods rapid prediction of K-value in fish. Vib. Spectrosc..

[36] Grassi S., Casiraghri E., Alamprese C. (2018). Handheld NIR device: A non-targeted approach to assess authenticity of fish fillets and patties. Food Chem..

[37] Currò S., Savini F., Fasoleto L., Indio V., Tomasello F., Rampazzo G., Zironi E., Pagliuca G., Gazzotti T., Prandini L. (2025). Application of near-infrared spectroscopy as at line method for the evaluation of histamine in tuna fish (*Thunnus albacares*). Food Control.

[38] Cheng J.H., Sun D.W. (2017). Partial least squares regression (PLSR) applied to NIR and HSI spectral data modeling to predict chemical properties of fish muscle. Food Eng. Rev..

[39] Khoshnoudi-Nia S., Moosavi-Nasab M. (2019). Prediction of various freshness indicators in fish fillets by one multispectral imaging system. Sci. Rep..

[40] Sanhueza M.I., Montes C.S., Sanhueza I., Montoya-Gallardo N.I., Escalona F., Luarte D., Escribano R., Torres S., Godoy S.E., Amigo J.M. (2024). VIS-NIR hyperspectral imaging and multivariate analysis for direct characterization of pelagic fish species. Spectrochim. Acta Part A Mol. Biomol. Spectrosc..

